# Why Increased Focus on Aging With Disability Matters to Gerontology Research, Policy, and Practice

**DOI:** 10.1093/geroni/igaa057.2212

**Published:** 2020-12-16

**Authors:** Margaret Campbell, Matthew Janicki

## Abstract

This symposium elaborates on the theme of the 2020 conference, “Why Age Matters”, to include aging with disability. We ask: “can an increased focus on aging with disability within gerontological research, policy, and practice advance our knowledge of disablement across the life cycle and improve our design and implementation of health and social service interventions’? Five experts will address this from differing perspectives (including gerontology and rehabilitation). One presentation draws on national/ regional data to illustrate the changing demographics of aging and disability and highlights the health consequences of aging with- and aging into, long-term physical disabilities. A second uses data from a mixed methods study to demonstrate the unique challenges experienced by adults aging with spinal cord injury with a focus on the impact of specific environmental barriers and facilitators to maintain health and participation in social roles. A third covers three reports on data from a scoping review to document the exclusion of middle-aged and older adults with disabilities from behavioral clinical trials and describes how translational research strategies can be used to help close this gap. A fourth presents examples of how technologies, such as videoconferencing and voice activation, are being used to deliver and enhance existing EB interventions to improve health, physical activity, and participation for individuals aging with mobility impairments. The last one draws on research and scholarly work from both gerontology and rehabilitation to highlight the co-occurring issues of ageism and ableism and describes how reducing ableism is central to successfully reframing aging. Lifelong Disabilities Interest Group Sponsored Symposium.

